# Surveillance of Intra-Abdominal Pressure and Intestinal Barrier Function in a Rat Model of Acute Necrotizing Pancreatitis and Its Potential Early Therapeutic Window

**DOI:** 10.1371/journal.pone.0078975

**Published:** 2013-11-14

**Authors:** Wei-Dong Li, Lin Jia, Ya Ou, Yao-Xing Huang, Shu-man Jiang

**Affiliations:** 1 Department of Gastroenterology, Guangzhou First People's Hospital, Guangzhou Medical University, Guangzhou, Guangdong Province, China; 2 Department of Gastroenterology, Guangzhou Nansha Central Hospital, Guangzhou, Guangdong Province, China; University of Szeged, Hungary

## Abstract

**Objectives:**

To monitor intra-abdominal pressure (IAP) and intestinal barrier function in a rat model of acute necrotizing pancreatitis (ANP) to elucidate a potential relevant therapeutic window.

**Methods:**

Sprague-Dawley rats were randomly divided into experimental or control groups. The ANP group (n = 40) was injected with 4.5% sodium taurocholate into the pancreatic duct to induce ANP. The controls received only abdominal opening surgery (sham-operated, SO; n = 40) or no treatment or surgery (baseline; 0 h, n = 20). The SO and ANP groups were then randomly subdivided into 3, 6, 12 and 24 h groups (n = 10 each). IAP was measured at each time point and the rats were sacrificed to measure the weight of accumulated ascites fluid and the amylase, endogenous creatinine (Cr), total bilirubin (TB), tumor necrosis factor- alpha (TNF-alpha), diamine oxidase (DAO), and D-lactate. Mortality and the development of pathological changes in the pancreas and intestines were also monitored.

**Results:**

IAP showed a continuous upward trend in the ANP group, with values 2 to 3 times higher than those in the SO group at the corresponding time points and the rising rate was peaking at 6 h. The levels of plasma amylase, TNF-alpha, Cr, TB, DAO, and D-lactate also gradually increased in the ANP group over time and were significantly higher than in the SO group at 3, 6, 12 and 24 h (all P<0.05). Moreover, the rising rate of TNF-alpha, DAO, and D-lactate also peaked at 6 h.

**Conclusions:**

The ANP-induced changes in IAP, inflammatory factors and intestinal barrier that we observed in the rat model were especially obvious at 6 h post-induction, suggesting an early therapeutic window for the treatment of ANP in humans.

## Introduction

ANP is a common acute abdominal disease, with rapid onset and progression and high mortality [1]–[2]. An increase in inflammatory mediators and cytokines in the bloodstream has been shown to be associated with the initiation of ANP and the development multiple organ dysfunction syndrome (MODS) [3]–[4]. Intestinal microcirculation dysfunction, loss of intestinal barrier integrity, and extensive inflammatory processes in the retro peritoneum and visceral edema caused by massive fluid retention increase the risk of developing abdominal compartment syndrome (ACS). It has recently been proposed that intra-abdominal hypertension (IAH) and ACS may have been the cause of significant morbidity and mortality during the past decade [5], and several recent studies suggest that the development of IAH/ACS in the early stages of ANP is an important contributor to severe inflammatory response and MODS [6]–[7]. Therefore, an in-depth study of the development of intra-abdominal pressure (IAP) may greatly influence our ability to therapeutically postpone the development of ANP and reduce mortality. On the base of our previous experiments [8]–[9], we monitored IAP and other related disease indicators in a rat model of acute necrotizing pancreatitis (ANP), and aim to propose the concept of an early therapeutic window for ANP in this study.

## Materials and Methods

### Experimental animals and reagents

All the experimental procedures were performed in accordance with the guide for the care and use of laboratory animals (National Institutes of Health Publication no. 86–23, revised 1985) and were approved by the Guangzhou First People's Hospital ethics committee. Specific-pathogen-free grade female Sprague-Dawley rats (100) weighing between 200 and 230 g were purchased from the Experimental Animal Center of Guangzhou University of Chinese Medicine (license number: SCXK 2008–0020).

### Measurement parameters

MODS occur simultaneously or progressively following severe infection, severe trauma and shock. The affected organ systems involved are respiratory, cardiovascular, renal, hepatic, gastrointestinal, hematological, endocrine, and central nervous system. In our study, we have seen that dysfunction or failure of multiple organs or systems happened simultaneously or sequentially.

Endogenous creatinine (Cr) is secreted by glomerular filtration. It is a small molecular substance, and it can be through the glomerular filtration. When kidney function is injury, the accumulation of Cr in the body becomes harmful toxins. Therefore, creatinine is one of clinical common good renal function test. Total bilirubin (TB) is mainly used to diagnosis for liver disease or biliary if an exception occurs. In most of the liver and gallbladder diseases, TB can have varying degrees of increase. So TB is one of sensitive indexes of liver function damage. TNF alpha likely plays both pro- and anti-inflammatory roles in regulating pancreatic inflammation. TNF-alpha has increasing release in early stage of ANP course. Such increase plays a central role in the pro-inflammatory factor. When the intestinal barrier is damaged, diamine oxidase (DAO) and D-lactate are transferred into the blood circulation through the damaged mucosa in the early stage. So, the detection of plasma levels of DAO and D-lactate can promptly reflect the extent of damage and permeability changes in the small intestine, and they are the specificity and sensitivity indicators of intestinal barrier function evaluation [10].

### ANP induction

Rats were randomly divided into sham-operated (SO) and ANP groups (n = 40 each) and then randomly subdivided into 3, 6, 12 and 24 h groups, with 10 rats in each group. Rats of the ANP group were injected with 4.5% sodium taurocholate into the pancreatic duct to induce ANP. Rats of SO groups received abdominal surgery only. Another 20 rats served as the baseline control (0 h) and received no sodium taurocholate or surgery.

Prior to starting the experiments, all animals were given free access to food and water. The room was maintained on a 12 h light–dark cycle and at a temperature of 24°C. Food, but not water, was withdrawn 12 h before the experiment. ANP was induced by the modified Aho's method [11]–[12] and described by Huang and Li et al [8]–[9]. Intra-peritoneal injection of 10% chloral hydrate (400 mg/kg; Guangdong herbal medicine pharmaceutical chain co., LTD) was used for abdominal cavity anesthesia. Sodium taurocholate solution (4.5%; 1 ml/kg; Sigma-Aldrich (Saint Louis, MO, USA)) was administered into the common biliopancreatic duct of the ANP group by retrograde injection. The abdominal surgery of the SO group consisted of opening the abdomen, flipping the pancreas and duodenum 180°, returning them to their original position, then closing the abdominal cavity with two layers of sutures. All of the rats were then injected with normal saline (20 ml/kg) through the vena caudalis to supplement the loss of fluid during the surgery.

### Determination of IAP and weight of ascites fluid

Rats were anesthetized by intra-peritoneal injection of 10% chloral hydrate, placed in a supine position. A catheter was inserted through the lower left quadrant of the abdomen wall guided by a 20 gauge needle. The catheter was connected to the testing system. The testing system was composed of signal amplifer, digital voltmeter, data acquisition unite and computer. The BL-420E+ Biological Function experimental system (Chengdu TME Technology Co., Ltd., Chengdu, China) in the computer was used to determine IAP. After measuring IAP, the ascites fluid inside the abdominal cavity was absorbed onto pre-weighed cotton balls and weighed.

### Determination of plasma levels of biochemical indicators

Blood samples collected from the inferior vena cava, were misced with EDTA-2K anticoagulative liquor, then centrifuged at 1409×g for 10 minutes and stored at −70°C. Plasma amylase, Cr and TB levels were detected by a fully automatic biochemistry analyzer (AU5400; Olympus Corporation, Tokyo, Japan). The reagent of TB detection was Bilirubin Auto Total FS which from DiaSys Diagnostic Systems GmbH, Germany. The reagent of Cr detection was CRE which from Saint Louis, MO, USA. The levels of TNF-alpha, DAO and D-Lactate were determined by specific enzyme-linked immunosorbent assay kits (Mizuno Bio Corporation, Taiwan, China).

### Histopathology

Pancreas and small intestine (at about 10 cm from pyloric sphincter) samples were collected, fixed in 10% formalin and embedded in paraffin, and then 4 µm sections were cut and stained with hematoxylin and eosin. The pathological changes in the pancreas and small intestine were evaluated by a professional pathologist according to the Schmidt pancreas score [13] and Chiu's small intestine score systems [14].

### Statistical analysis

All measurements were expressed as the mean ± the standard error of the mean (SEM), and were compared by two-way ANOVA test for multiple comparisons by using SPSS 13.0 for Windows. The p-values of multiple comparisons were adjusted to control the type I error rate. P<0.05 was considered statistically significant.

## Results

### IAP levels and weight of ascites fluid

The ascites fluid from the ANP-induced rats appeared kermesinus, but amber in the SO controls. Rats in the baseline group contained only small amounts of amber fluid. A gradual upward trend in the weight of ascites fluid over time was observed in the ANP groups. There was an obvious increase in the first 12 h, (all *P*<0.05), and a slight additional increase in the next 12 h (*P*>0.05; Table 1). Furthermore, the weight of ascites fluid was significantly greater in the ANP groups than in the SO groups at corresponding time point (all *P*<0.05).

**Table 1:** Data of weight of ascites, mortality, pathological scores of intestine and pancreas for groups at each time point.

Time point (h)	Group	Weight of ascites (g)	Mortality	Pathological score of intestine	Pathological score of pancreas
0 h		0.7(0.4)	0%	1.5(0.5)	1.96(0.45)
3 h	SO	1.2(0.3)	0%	1.7(0.3)	2.25(0.38)
	ANP(N = 10)	4.6(0.8) ^a^	0%	3.7(0.05) ^a^	5.6(0.12) ^a^
6 h	SO	1.5(0.7)	0%	1.6(0.52)	1.5(0.2)
	ANP(N = 8)	5.8(1.5)^ab^	20%	7.81(1.1)^ab^	7.26(0.52) ^ab^
12 h	SO	0.8(0.4)	0%	2.2(0.3)	2.56(0.17)
	ANP(N = 8)	8.8(1.3)^abc^	20%	11.1(0.8)^abc^	11.8(0.39) ^abc^
24 h	SO	2.2(0.3)	0%	2.2(0.4)	2.0(0.23)
	ANP(N = 6)	10.5(2.6)^abc^	40%	13.8(0.79)^abcd^	14.10(0.22) ^abcd^

In ANP and SO, ^a^ *P*<0.05 vs 0 h;^b^ *P*<0.05 vs 3 h; ^c^ *P*<0.05 vs 6 h; ^d^ *P*<0.05 vs 12 h.

There were significantly differences between ANP and SO at each time point.

Data for each group at each time point (mean (SEM))

**Figure 1:**
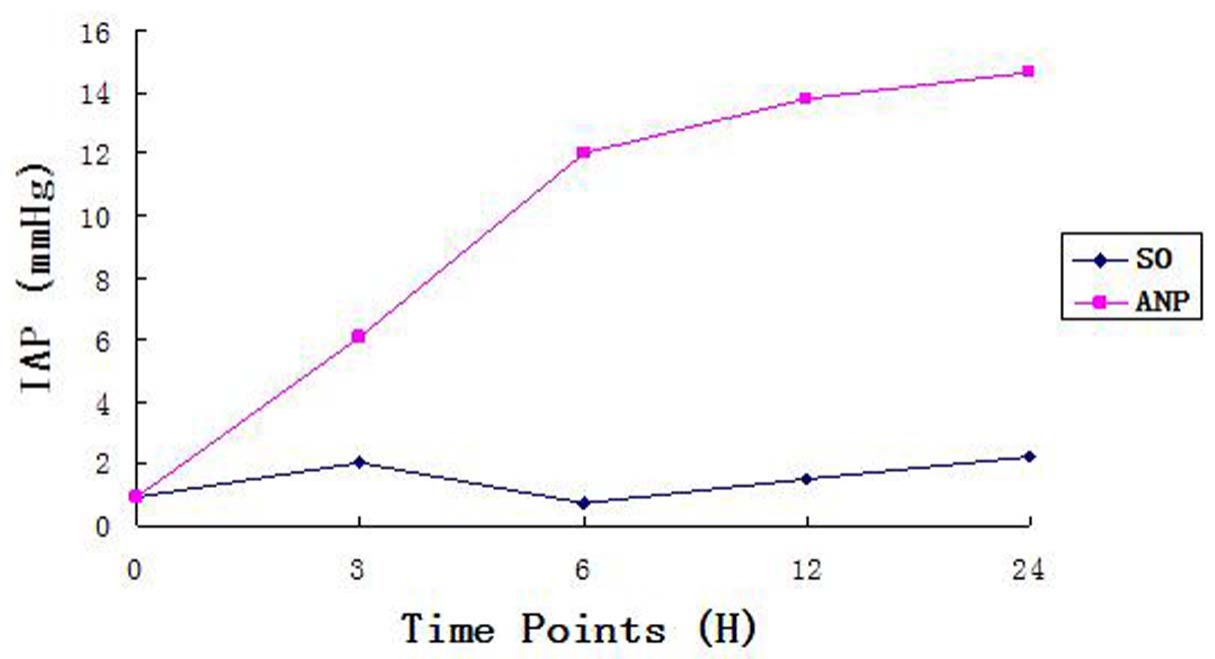
Induction of IAP. Described the variation tendency of IAP in two groups.

As shown in Figure 1, both the ANP and SO groups exhibited a continuous increase in IAP over time. In the ANP group, IAP increased dramatically up to 12 h (P<0.05), the rising rate was peaking at 6 h, then continued to rise slightly, but significantly (P<0.05), until the end of the experiment (24 h). Compared with the ANP groups, IAP was 2 to 3 times lower in the SO groups at the corresponding time points (all P<0.05). In addition, the IAP of the ANP groups was significantly higher at 3, 6, 12, and 24 h than at the baseline (0 h), whereas there was no apparent difference between the SO groups and the baseline.

### Mortality and Pathological severity scores

The pathological severity scores for the pancreas were dramatically higher in the ANP groups than in the SO groups (all *P*<0.05) and gradually increased over time. The pancreas severity scores were higher in the ANP groups at 3, 6, 12, and 24 h than in the baseline control, whereas no obvious difference was observed between the SO groups and baseline (Table 1).

As shown in Table 1, the intestine pathological severity score also increased in the ANP group over time, most significantly at 6 h, and scores were significantly higher in the ANP groups than in the SO groups at the corresponding time points (all *P*<0.05).

Mortality in the ANP group gradually increased over time, whereas, no mortality was observed in the SO groups (Table 1). At the end of the experiment, ANP 3 h group n = 10, ANP 6 h group n = 8, ANP 12 h group n = 8, ANP 24 h group n = 6.

Levels of plasma amylase, TNF-alpha, Cr,TB,DAO, and D-lactate.

**Figure 2:**
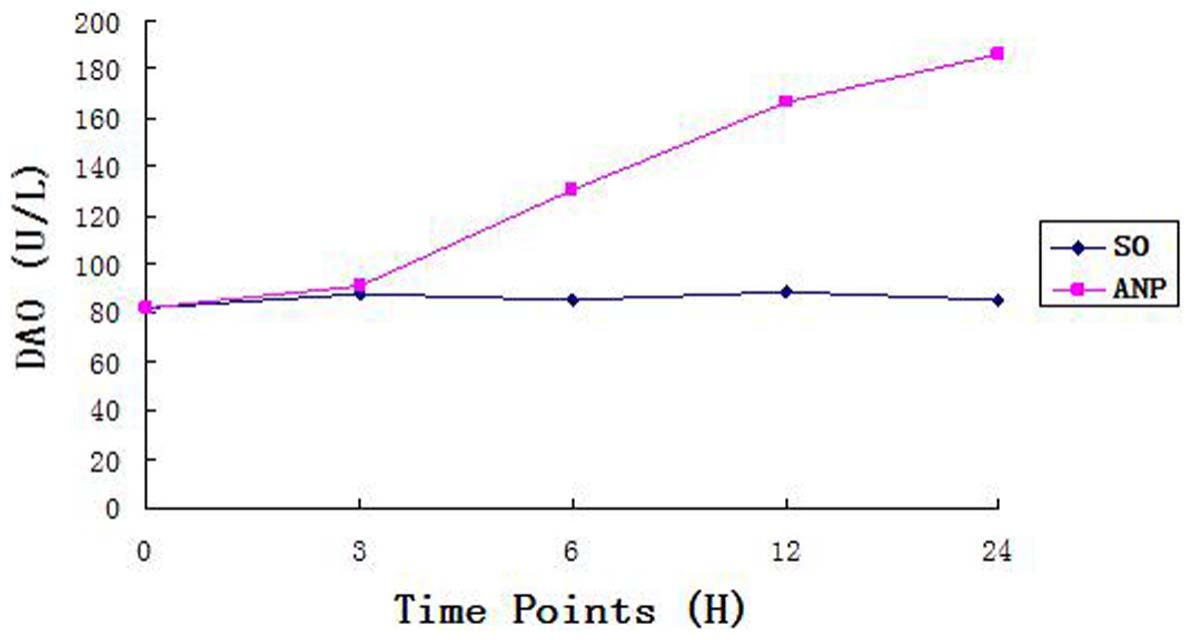
Induction of DAO. Described the variation tendency of DAO in two groups.

**Figure 3:**
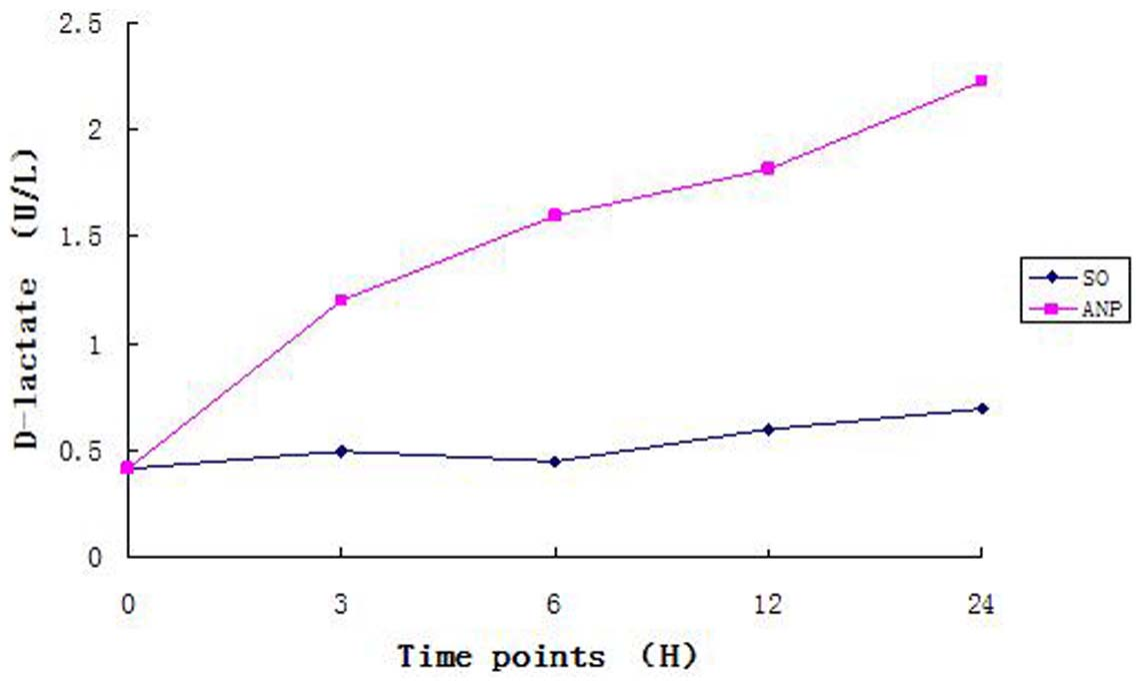
Induction of D-Lactate. Described the variation tendency of D-Lactate in two groups.

The levels of plasma amylase, TNF-alpha, Cr,TB,DAO and D-lactate rose continuously over time in the ANP group, the rising rate of TNF-alpha, DAO, and D-lactate were peaking at 6 h (Table 2 and Figure 2, 3). Moreover, all these indexes were higher in the ANP groups than in the SO groups at each time point (all *P*<0.05). These plasma biochemical indicators were also higher in the ANP groups at 3, 6, 12 and 24 h time points than in the baseline control, but no significant difference was seen between the SO groups and the baseline.

**Table 2:** Data of amylase, Cr (endogenous creatinine), TB (total bilirubin) and TNF-alpha for groups at each time point.

Time point (h)	Group	Amylase (U/L)	TB (µmol/L)	Cr (µmol/L)	TNF-alpha (pg/ml)
0 h		305.5(137.2)	0.2(0.07)	32.0(5.6)	6.3(2.7)
3 h	SO	357.9(39.5)	0.91(0.38)	30.5(6.0)	13.5(1.4)
	ANP(N = 10)	3624.8(1411.3) ^a^	5.36(1.74) ^a^	80.2(33.8) ^a^	80.7(18.3) ^a^
6 h	SO	596.2(29.5)	0.82(0.18)	35.5(4.9)	8.9(0.4)
	ANP(N = 8)	8930.8(1853.1) ^ab^	6.36(1.90) ^ab^	98.2(42.8) ^ab^	166.2(28.6)^ab^
12 h	SO	587.8 (62.9)	0.80(0.25)	29.5(8.2)	15.6(1.5)
	ANP(N = 8)	9767.5(2996.6) ^abc^	8.02(2.64) ^abc^	150.2(30.8) ^abc^	103.2(17.6)^abc^
24 h	SO	342.6(17.8)	0.91(0.38)	33.5(7.5)	11.6(6.9)
	ANP(N = 6)	11024.5 (2203.5) ^abcd^	8.36(3.74) ^abcd^	204.2(38.8) ^abcd^	107.3(20.2)^abcd^

In ANP and SO, ^a^ *P*<0.05 vs 0 h;^b^ *P*<0.05 vs 3 h; ^c^ *P*<0.05 vs 6 h; ^d^ *P*<0.05 vs 12 h.

There were significantly differences between ANP and SO at each time point.

Cr: endogenous creatinine, TB: total bilirubin.

ANP 3 h group n = 10, ANP 6 h group n = 8, ANP 12 h group n = 8, ANP 24 h group n = 6.

Data for each group at each time point (mean (SEM))

## Discussion

ANP is a common dangerous clinical emergency with rapid progression and a fatality rate as high as 30% [9], [15]–[16]. At the early stages of ANP, severe inflammation leads to blood capillary permeability, an increase in intra- abdominal drainage, edema of peripancreatic and retroperitoneal tissues and necrosis in many tissues. At the later stages of ANP, many factors (such as excessive protein loss) lead to abdominal wall edema and loss of integrity, which results in the pathological enlargement of intra-abdominal organs [17]–[21]. Fluid retention caused an the increase in extracellular fluid volume, together with intestinal paralysis, leads to a rise in IAP and the production of bloody ascites fluid; ultimately leading to ACS. The clinical manifestations of ACS are severe abdominal distension, ventilatory impairment, refractory hypercapnia, and liver and kidney dysfunction. De Waele et al. [22] measured IAPs higher than 15 mmHg in 21 out of 27 SAP patients. Thus the timely and effective treatment of IAP/IAH in the early stages of disease may be the key to preventing development of MODS and to improving the cure rate. Early mitigation of IAP has not been reported as an approach to treat ANP and this study is the first to put forward the concept of an early therapeutic window.

Normal IAP levels range from 5 to 7 mmHg. IAH is defined as a sustained or repeated pathological elevation in IAP≥12 mmHg. IAH is graded as follows: grade I: IAP 12–15 mmHg, grade II: IAP 16–20 mmHg, grade III: IAP 21–25 mmHg, grade IV: IAP>25 mmHg. ACS is defined as a sustained IAP>20 mmHg (with or without an APP<60 mmHg) that is associated with new organ dysfunction/failure. ACS is defined as a sustained IAP>20 mmHg that is associated with organ dysfunction/failure [21]. We inferred from these values, that IAH would be induced in the ANP group in our study when the IAP was more than two times higher than that of SO group. In fact, the IAP values detected in the ANP group at 3, 6, 12 and 24 h after induction, were more than 4 times higher than those of SO group at each time point. The inflammatory cytokine TNF-alpha and intestinal barrier function exhibited the same trend as IAP in the course of ANP development. In particular, we found that 6 h after disease induction was a critical time point in the course of ANP, and that rapid deterioration began after this time point. These results suggest that ANP in humans may deteriorate to MODS if no intervention is administered within the first 6 h. The combination of IAH, TNF-alpha induction and intestinal barrier dysfunction can lead to systemic capillary leakage then to humeral regulation disequilibrium, which together with severe inflammation and other factors can eventually lead to the ACS. ACS, in turn, further decreases liver and kidney function and exacerbates pancreatic microcirculation dysfunction.

Therefore, we think within 6 h of ANP onset may be the key timeframe for therapeutic interventions, such as early containment of IAP increasing, inhibition of the release of inflammatory factors and improvement of intestinal barrier function and suggest the potential early therapeutic window for the treatment of ANP in human. These approaches will be a primary focus in our future therapeutic studies of ANP patients.
